# Scoring System for Mortality in Patients Diagnosed with and Treated Surgically for Differentiated Thyroid Carcinoma with a 20-Year Follow-Up

**DOI:** 10.1371/journal.pone.0128620

**Published:** 2015-06-26

**Authors:** David López-Bru, Antonio Palazón-Bru, David Manuel Folgado-de la Rosa, Vicente Francisco Gil-Guillén

**Affiliations:** 1 Department of Otorhinolaryngology, General University Hospital, Elda, Alicante, Spain; 2 Department of Clinical Medicine, Miguel Hernández University, San Juan de Alicante, Alicante, Spain; 3 Research Unit, General University Hospital, Elda, Alicante, Spain; Seoul National University, REPUBLIC OF KOREA

## Abstract

**Background:**

Differentiated thyroid carcinoma (DTC) is associated with an increased mortality. Few studies have constructed predictive models of all-cause mortality with a high discriminating power for patients with this disease that would enable us to determine which patients are more likely to die.

**Objective:**

To construct a predictive model of all-cause mortality at 5, 10, 15 and 20 years for patients diagnosed with and treated surgically for DTC for use as a mobile application.

**Design:**

We undertook a retrospective cohort study using data from 1984 to 2013.

**Setting:**

All patients diagnosed with and treated surgically for DTC at a general university hospital covering a population of around 200,000 inhabitants in Spain.

**Participants:**

The study involved 201 patients diagnosed with and treated surgically for DTC (174, papillary; 27, follicular).

**Exposures:**

Age, gender, town, family history, type of surgery, type of cancer, histological subtype, microcarcinoma, multicentricity, TNM staging system, diagnostic stage, permanent post-operative complications, local and regional tumor persistence, distant metastasis, and radioiodine therapy.

**Main outcome measure:**

All-cause mortality.

**Methods:**

A Cox multivariate regression model was constructed to determine which variables at diagnosis were associated with mortality. Using the model a risk table was constructed based on the sum of all points to estimate the likelihood of death. This was then incorporated into a mobile application.

**Results:**

The mean follow-up was 8.8±6.7 years. All-cause mortality was 12.9% (95% confidence interval [CI]: 8.3–17.6%). Predictive variables: older age, local tumor persistence and distant metastasis. The area under the ROC curve was 0.81 (95% CI: 0.72–0.91, p<0.001).

**Conclusion:**

This study provides a practical clinical tool giving a simple and rapid indication (via a mobile application) of which patients with DTC are at risk of dying in 5, 10, 15 or 20 years. Nonetheless, caution should be exercised until validation studies have corroborated our results.

## Introduction

Thyroid cancer is relatively uncommon, though it is still the most frequent malignant endocrine tumor in our organism [1,2]. Differentiated thyroid carcinoma involves papillary or follicular carcinoma. Papillary carcinoma is the most common of all malignant thyroid tumors, followed by follicular carcinoma [3,4]. In addition, differentiated thyroid carcinoma is associated with a significant increase in mortality [5]. This may explain why some authors have analyzed all-cause mortality in patients with this type of cancer, evaluating both the incidence of mortality and the various associated factors in cohorts of patients diagnosed with differentiated thyroid carcinoma [3,5–20].

In Spain, as in other countries, the incidence of differentiated thyroid carcinoma is rising, especially in men, although it is more frequent in women [2,21]. Situated in the southeast of Spain, the Valencian Community has a population that increased from 3,722,362 inhabitants in 1984 to 4,987,017 inhabitants in 2013 [22]. All the oncological services in this Community are free to access and free of charge for all residents. Thyroid cancer is dealt with via a Committee of Endocrine and Thyroid Tumors. The best therapeutic option is decided after discussion of each individual case [23].

Analysis of hospital registries in the Valencian Community recorded over 30 years has determined the all-cause mortality of patients diagnosed with and treated surgically for differentiated thyroid carcinoma. Unlike other studies [3,5–20], which only addressed mortality incidence and its associated factors, this study constructed a model able to predict all-cause mortality up to a maximum period of 20 years. The model is based on a scoring system that has been adapted for use as a mobile telephone application and which can be applied in daily clinical practice for the simple determination of which patients are more likely to die during this 20-year period. With this knowledge we can undertake a stricter follow-up of patients who have a higher likelihood of dying.

## Methods

### Study population

The study population comprised all patients diagnosed with and treated surgically for differentiated thyroid carcinoma by the Department of Health in Elda. This area is an industrial zone in the Valencian Community with a medium-low economic and cultural level. The number of inhabitants in this Department ranged from 164,999 in 1984 to 193,588 in 2013 [22].

### Study design and participants

This retrospective cohort study covered 30 years (1984–2013) and included all patients diagnosed with and treated surgically for differentiated thyroid carcinoma. A patient was considered to have this disorder if the diagnosis was confirmed histologically. To determine this diagnosis, an examination was undertaken of all clinical records of patients who underwent surgery for any type of thyroid disorder. The records were examined in all the databases of any medical-surgical service possibly related with patients diagnosed with differentiated thyroid carcinoma (Otorhinolaryngology, General Surgery, Oncology, Endocrinology, Pathology, Radiology, Committee of Endocrine and Thyroid Tumors, Clinical Documentation, and Hospital Discharge Registry). Information was obtained about the time between diagnosis of differentiated thyroid carcinoma and date of death, or date of end of study (December 31, 2013), or date of last clinical contact (in those cases where we were unaware of whether the patient had died during the follow-up).

### Variables and measurements

The main outcome variable was all-cause mortality during the follow-up period. This was assessed by analyzing the databases mentioned above and in case of doubt by telephoning the number recorded on the hospital records (land line and mobile).

The following secondary variables were also recorded at diagnosis: age, gender, number of inhabitants in the town of residence (</≥ 10,000 inhabitants) [22], known family history of thyroid cancer, type of thyroid surgery (total 1–2 stages, partial or none), type of cervical lymph node surgery (central+lateral, central, lateral, or none), type of differentiated cancer (papillary or follicular), histological subtypes (good or poor prognosis) [24], solitary papillary intraglandular microcarcinoma <1 cm (yes or no), multicentricity (solitary or multifocal), adenopathy with a histopathological diagnosis (central+lateral, central, lateral, or none), AJCC (American Joint Committee on Cancer) TNM staging system [25], diagnostic stage (I, II, III, IVa, IVb or IVc), permanent postoperative recurrent nerve paralysis (yes or no), permanent postoperative hypoparathyroidism (yes or no), local tumor persistence (persistence of diseased thyroid tissue in the surgical thyroid bed in patients who had previously received primary surgical treatment) and regional lymph node persistence (persistence of cervical and/or upper mediastinal lymph node disease in patients who had previously been treated with cervical lymph node surgery) (yes or no), distant metastasis (yes or no), and radioiodine therapy (yes or no). These variables were all obtained from the sources of information mentioned above, except for the population of the town of residence, which was obtained from official data by the National Statistics Institute [22], and the stage, which was obtained from the 2010 AJCC classification (7th edition) [25], using the TNM parameters, type of cancer and age, obtained as described above.

### Sample size

The sample comprised 201 patients, of whom 26 died. Thus, using a 95% confidence interval and with an expected area under the ROC curve (AUC) of 0.8, in order to contrast an AUC different to 0.5 the power obtained was 99.85% [26].

### Statistical analysis

Absolute and relative frequencies were used to describe the qualitative variables, with means and standard deviations for the quantitative variables. A Cox multivariate regression model was constructed to determine which variables were associated with all-cause mortality and the adjusted hazard ratios (HR) were obtained. As we had few patients, we selected a maximum number of explanatory variables in the model (one for each 50 *non deaths*). For this we used a stepwise algorithm to determine those variables that might predict mortality better. To obtain the variables in the model we analyzed all the possible combinations with a maximum of 3 variables (4,525), calculating the value of the C-statistic in all of them. The combination with the highest value was then selected. The goodness of fit of the model was assessed by the score test. From the β coefficients of the multivariate model a risk table was constructed based on the sum of the points to estimate the probability of death [27]. After calculating the points and their associated risk, the AUC was calculated for this mortality scale and the following cut points were selected on the scale constructed: 1) *Optimum*: that which minimized the square root of (1-Sensitivity)^2^ + (1-Specificity)^2^; 2) *Confirmation*: the minimum score that had a positive likelihood ratio (PLR) ≥10; 3) *Discard*: the maximum score that had a negative likelihood ratio (NLR) <0.1. Points 2 and 3 were chosen because, according to the Evidence Based Medicine School, they are those that permit the positivity of a diagnostic test to be confirmed or discarded conclusively [28]. Using these cut points, we calculated the indicators of validity (sensitivity and specificity), yield (positive predictive value (PPV) and negative predictive value (NPV)) and usefulness (PLR and NLR). The risk groups for death were also designed using the cut points obtained previously: low risk (less than the *discard point*), medium risk (from the *discard point* to the *optimal point*), high risk (from the *optimal point* to the *confirmation point*), and very high risk (equal to or greater than the *confirmation point*). Finally, the survival of these groups was compared using the log-rank test. The survival curves were represented using the Kaplan-Meier technique. All the analyses were done with an α = 5% and the associated confidence interval (CI) was calculated for each relevant parameter. All the analyses were done with IBM SPSS Statistics 19.

### Mobile application

The model was implemented on a mobile telephone application (App) (S1 Text). Using this tool the likelihood of all-cause death can be calculated without the need for mathematical calculations. This App is available for the following mobile telephone operating systems: Android and Apple.

### Ethical questions

This study was approved by the Ethics Committee of the Department of Health of Elda. Given that the study was undertaken using retrospective data, no informed consent was requested from the patients. The Ethics Committee approved this procedure.

## Results

During a mean follow-up of 8.8±6.7 years, of 201 patients with differentiated thyroid carcinoma, 26 died (12.9%, 95% CI: 8.3–17.6%). This corresponds to an incidence density of 15 deaths per 1,000 person-years (95% CI: 10–22 deaths per 1,000 person-years). The causes of death were: thyroid cancer (7), other cancers (7), cardiovascular or respiratory diseases (8), diabetes-related problems (1), and unknown but with no relation to the thyroid cancer (3).

Table 1 shows the descriptive and analytical characteristics of the study sample. Regarding the descriptive characteristics, the mean age was 46.6 years, there were more women (82.1%) and more cases of papillary carcinoma (86.6%). Furthermore, the prevalence of local tumor persistence was 6.0% and the proportion with distant metastasis at diagnosis was 1.5%. Concerning the tumor stage, the highest prevalence was stage I (69.2%), followed by stages III (12.4%) and IVa (10.9%). None of our patients had stage IVb cancer. Notably, over 90% of the patients were treated with radioiodine therapy (91.5%).

**Table 1:** Descriptive characteristics and hazard ratios for predicting mortality in patients with differentiated thyroid cancer.

	Total		
	n = 201	HR for all-cause mortality	
Variable	n(%)/x±s	(95% CI)	p-value
**Age (years)**	46.6±15.0	1.11(1.07–1.15)	<0.001
**Male gender**	36(17.9)	N/M	N/M
**Living in city**	182(90.5)	N/M	N/M
**Known family history of thyroid cancer**	21(10.4)	N/M	N/M
**Thyroid surgery:**		N/M	N/M
**Total (1 time)**	162(80.6)		
**Total (2 times)**	31(15.4)		
**Partial**	8(4.0)		
**Cervical lymph node surgery:**		N/M	N/M
**Central+Lateral**	42(20.9)		
**Central**	62(30.8)		
**Lateral**	3(1.5)		
**None**	94(46.8)		
**Papillary histology**	174(86.6)	N/M	N/M
**Histological subtype of favorable prognosis**	166(82.6)	N/M	N/M
**Solitary papillary intraglandular microcarcinoma <1 cm**	47(23.4)	N/M	N/M
**Solitary**	120(59.7)	N/M	N/M
**T:**		N/M	N/M
**T1**	120(59.7)		
**T2**	42(20.9)		
**T3**	35(17.4)		
**T4**	4(2.0)		
**N:**		N/M	N/M
**N0**	133(66.2)		
**N1a**	28(13.9)		
**N1b**	40(19.9)		
**M1**	3(1.5)	114.32(13.64–958.31)	<0.001
**Stage:**		N/M	N/M
**I**	139(69.2)		
**II**	12(6.0)		
**III**	25(12.4)		
**Iva**	22(10.9)		
**IVc**	3(1.5)		
**Permanent postoperative recurrent laryngeal nerve paralysis**	9(4.5)	N/M	N/M
**Permanent postoperative hypoparathyroidism:**	23(11.4)	N/M	N/M
**Local tumor persistence**	12(6.0)	3.30 (1.21–9.01)	0.020
**Lymph node persistence**	12(6.0)	N/M	N/M
**Radioiodine**	184(91.5)	N/M	N/M

n(%), absolute frequency(relative frequency); x±s, mean±standard deviation; HR, adjusted hazard ratio; CI, confidence interval; N/M, not in the multivariate model.

Goodness-of-fit of the model: Χ^2^ = 111.2, p<0.001, C-statistics = 0.836 (standard error = 0.065).

We obtained the following independent variables in our predictive model for death: older age (HR = 1.11, 95% CI: 1.07–1.15, p<0.001), local tumor persistence (HR = 3.30, 95% CI: 1.21–9.01, p = 0.020) and distant metastasis (HR = 114.32, 95% CI: 13.64–958.31, p<0.001).

**Fig 1:**
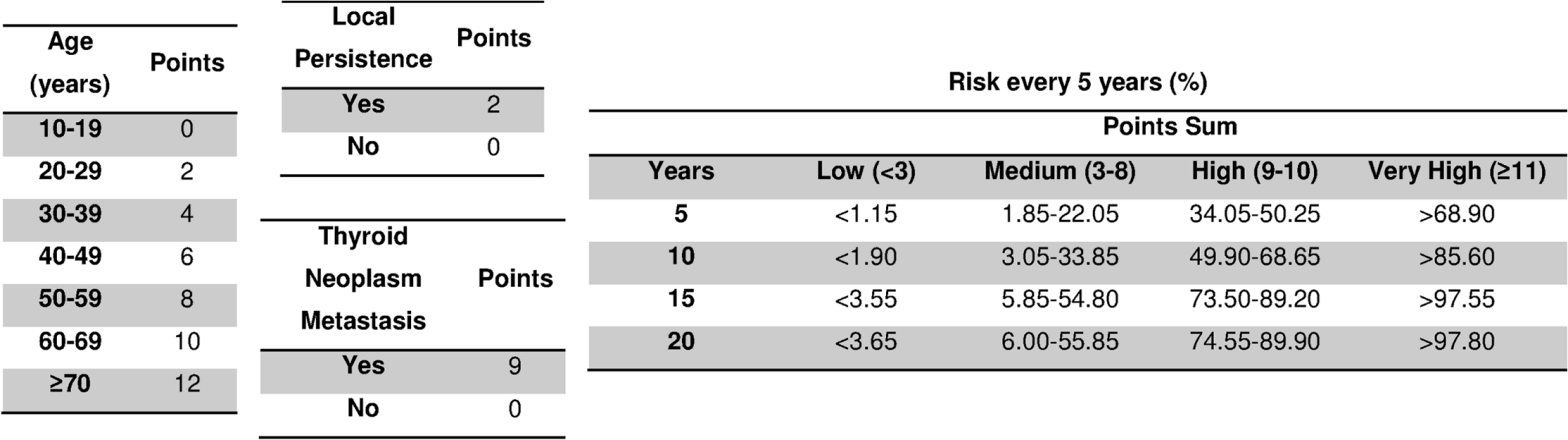
Predictive model for all-cause mortality in patients with differentiated thyroid carcinoma.

**Fig 2:**
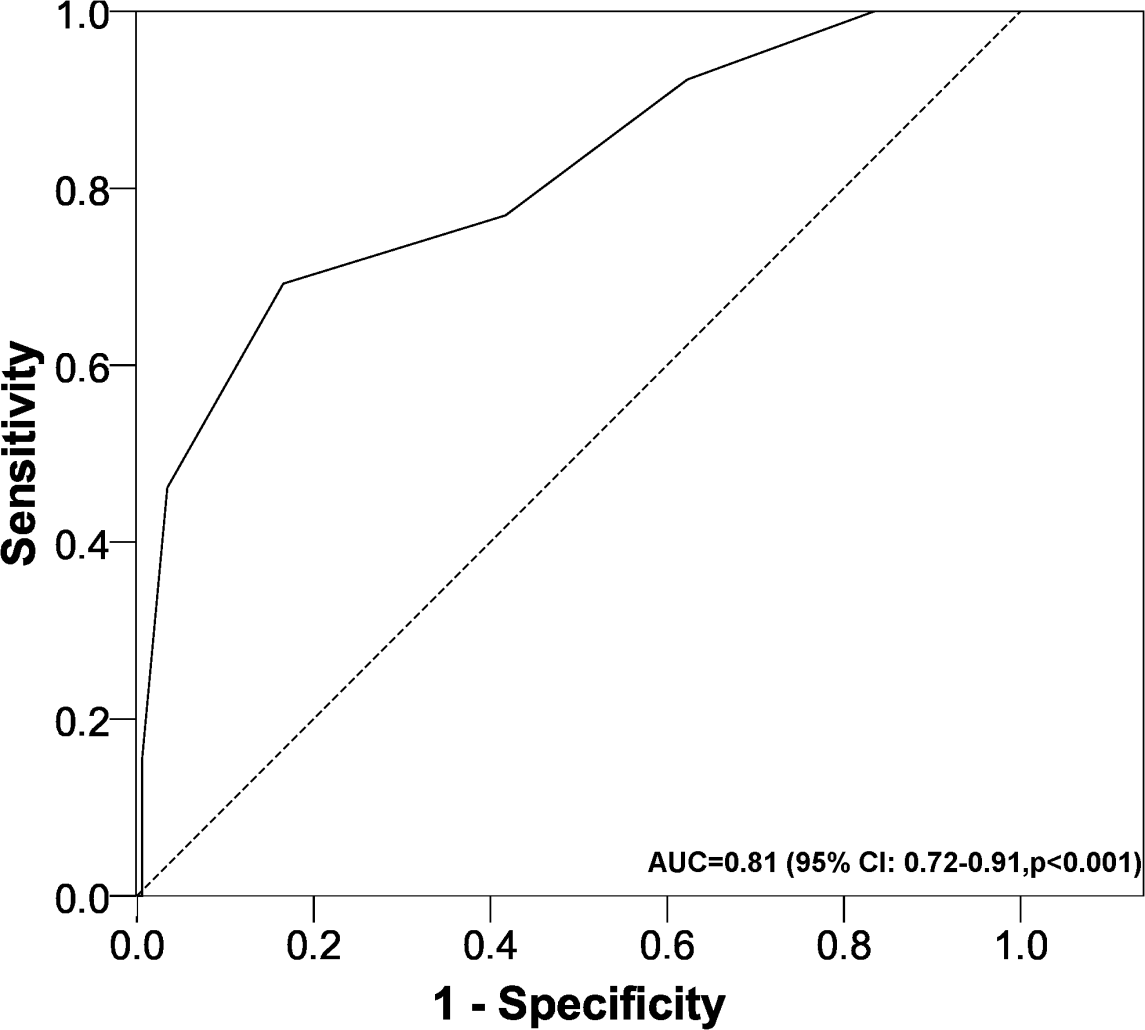
Area under the ROC curve of the predictive model. ROC, receiver operating characteristic; AUC, area under the curve; CI, confidence interval.

The multivariate model (Table 1) provided the predictive scale for mortality (Fig 1); this figure shows the scoring system with the risk groups for predicting mortality. This predictive model has an AUC (Fig 2) of 0.81 (95% CI: 0.72–0.91, p<0.001). The cut points (optimum, 9; confirmation, 11; discard, 3) and their indicators of validity (sensitivity and specificity), yield (PPV and NPV) and usefulness (PLR and NLR) are shown in Table 2.

**Table 2:** Cut points of the predictive model and their indicators of validity, yield and usefulness.

		Sensitivity	Specificity	PPV	NPV	PLR	NLR	
Point	Value	(95% CI)	(95% CI)	(95% CI)	(95% CI)	(95% CI)	(95% CI)	p-value
**Optimum**	9	0.69 (0.48–0.85)	0.83 (0.77–0.88)	0.38 (0.25–0.54)	0.95 (0.90–0.98)	4.18 (2.75–6.36)	0.37 (0.21–0.66)	<0.001
**Confirmation**	11	0.46 (0.27–0.66)	0.97 (0.92–0.99)	0.67 (0.41–0.86)	0.92 (0.87–0.96)	13.46 (5.53–32.75)	0.56 (0.39–0.80)	<0.001
**Discard**	3	1.00 (0.84–1.00)	0.17 (0.12–0.23)	0.15 (0.10–0.22)	1.00 (0.85–1.00)	1.20 (1.12–1.28)	0.00 (0.00-∞)	<0.001

PPV, positive predictive value; NPV, negative predictive value; PLR, positive likelihood ratio test; NLR, negative likelihood ratio test; CI, confidence interval.

**Fig 3:**
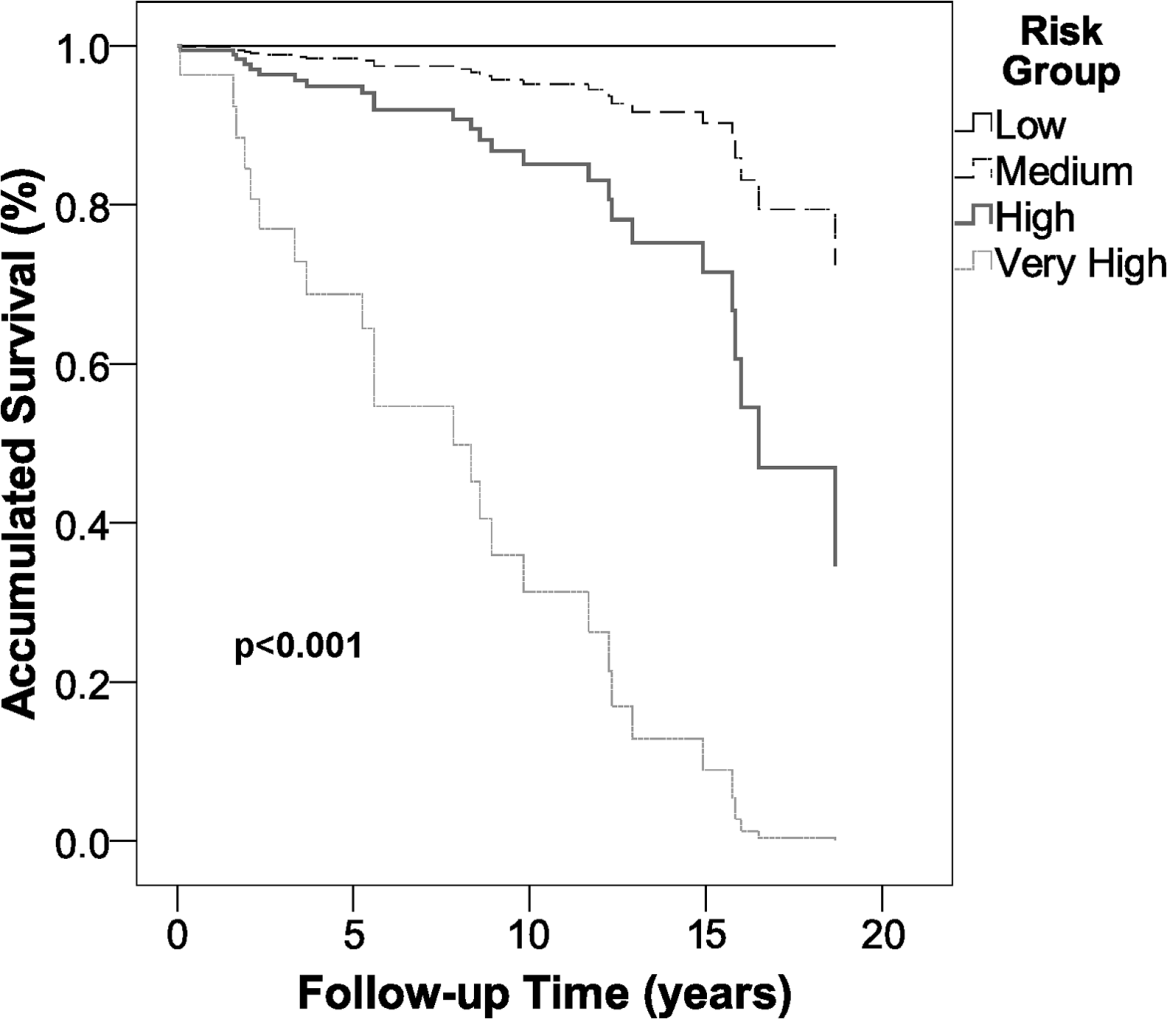
Survival of the different risk groups.

Fig 3 shows the differences in survival between the different risk groups (p<0.001). The survival can be seen to fall as the risk increases.

## Discussion

This analysis of all cases of differentiated thyroid carcinoma over 30 years in our healthcare area found that one in every eight persons died during a mean follow-up of almost 9 years. Other studies examining all-cause mortality in this type of patients involved different follow-up periods (ranging from 3.0 to 28.7 years), which makes it difficult to compare the magnitude of mortality found in this study. Probably, as the mean follow-up period in our study was within the range noted above, our magnitude of mortality was also within the interval reported by these other authors (2.5–55.0) [3,5–20].

Our predictive model showed that a patient who was older, with local tumor persistence and distant metastasis at diagnosis had a greater likelihood of all-cause mortality. The other studies [3,5–20] coincide regarding both age and metastasis. In addition, they found greater mortality associated with larger tumor size, extrathyroid extension, lymph node involvement, male gender, external radiotherapy, iodine radioisotopes, high levels of thyroglobulin, and certain histological subtypes. However, none of these other studies evaluated local tumor persistence at diagnosis. We think that patients who present this feature experience greater mortality because performing an incomplete excision of the thyroid tissue could affect mortality in differentiated thyroid carcinomas.

An overall analysis of the factors in the model shows that most have already been found to be risk factors [3,5–20]. However, we evaluated the model globally; i.e., considering the three factors together (age, local tumor persistence and distant metastasis). Local tumor persistence was a factor with a weight in mortality equivalent to 10 years of age, as can be seen in Fig 1. In other words, it is the factor with least weight in the predictive model. Nevertheless, the addition of the two points given by this parameter could mean a change in risk group, particularly if the patient is in the high-risk group, since if this patient has local tumor persistence he or she will automatically pass to the group with a very high risk.

After extensive validation, the results of this study provide daily clinical practice with a tool capable of predicting all-cause mortality in patients with differentiated thyroid carcinoma over 5, 10, 15 or 20 years simply and rapidly (mobile App), with a high discriminatory power. In addition, epidemiologically, the Evidence Based Medicine criteria [28] enable us to confirm or discard mortality within these time intervals conclusively. Thus, if a patient is in the low-risk group (NLR = 0.00), we can state confidently that this patient will not die during the period of time analyzed. On the other hand, if a patient is in the very high-risk group, we can confirm that this patient has a very high likelihood of dying. As it is not possible to modify this risk and given its great clinical relevance for the prognosis of our patients, we should stress the need to make an early diagnosis and perform radical surgery (total thyroidectomy) in order to reduce the added risk of age and local tumor persistence in our predictive model. This will enable the patient to be classified in a lower risk category, thereby improving the prognosis, as can be seen in Fig 3.

### Study strengths and limitations

The main strength of this study resides in the lack of other predictive models of all-cause mortality, although Sebastián et al. constructed a similar model classifying patients in risk groups [17]. However, the choice of these groups was based on survival quartiles (25%, 50% and 75%) rather than on risk groups that could conclusively confirm or discard the death of a patient. Furthermore, this model only encompassed half the prediction time of our study for high-risk patients. Likewise, our model generated an AUC above 0.80 with a high statistical power, despite involving a smaller sample size than that of other studies [3,9]. This confers our model with a high discriminating power. In addition, the methodology described herein could also be used to predict mortality in other types of cancer.

Although an important limitation of our study was that the sample size was just 201 patients, this was nevertheless sufficient for the aims of the study, as we were evaluating the predictive model and its associated AUC. The model was very significant (p<0.001) and the AUC very high (>80%). Furthermore, the number of variables in the model was selected bearing in mind our number of deaths and total patients and testing all the possible combinations. However, as occurred in the Framingham study, we obtained significant HR with wide CI. Nevertheless, as in the Framingham study, we were examining the overall model and not each individual variable [29–31]. Nevertheless, if the study had included more patients we could have introduced a greater number of variables in the model and divided the study sample into two parts in order to perform internal validation [32].

As we used data for a 30-year period we must accept a certain degree of measurement bias. However, to minimize this degree, all the sources of medical information (both computerized and non computerized) were strictly checked in an orderly manner.

Thus, after extensive validation, our models could be implemented in clinical practice. As did Hay et al. after their study [33], the authors of the present study encourage other researchers to determine the validity of our scoring system in other populations and then compare their predictive power with the current models.

## Conclusions

This study provides clinical practice with a tool giving simple rapid information (using a mobile application) about which patients with differentiated thyroid carcinoma are at risk of dying in 5, 10, 15 or 20 years. This tool also gives information about two risk groups for whom we can confirm or discard all-cause mortality conclusively. Nevertheless, we must be cautious as other studies are needed to validate and corroborate our results.

## Supporting Information

S1 Text Information about the mobile application. (DOC)

## References

[1] Hundahl SA, Fleming ID, Fremgen AM, Menck HR. A national cancer data base report on 53856 cases of thyroid carcinoma treated in the US, 1985–1995. Cancer. 1998; 83: 2638–2648. 9874472 10.1002/(sici)1097-0142(19981215)83:12<2638::aid-cncr31>3.0.co;2-1

[2] Davies L, Welch HG. Increasing incidence of thyroid cancer in the United States, 1973–2002. JAMA. 2006; 295: 2164–2167. 16684987 10.1001/jama.295.18.2164

[3] Yang L, Shen W, Sakamoto N. Population-based study evaluating and predicting the probability of death resulting from thyroid cancer and other causes among patients with thyroid cancer. J Clin Oncol. 2013; 31: 468–474. 10.1200/JCO.2012.42.4457 23270002

[4] Colonna M, Grande E, Jónasson JG, Eurocare Working Group. Variation in relative survival of thyroid cancers in Europe: results from the analysis on 21 countries over the period 1983–1994 (EUROCARE-3 study). Eur J Cancer. 2006; 42: 2598–2608. 16979888 10.1016/j.ejca.2006.04.026

[5] Eustatia-Rutten CF, Corssmit EP, Biermasz NR, Pereira AM, Romijn JA, Smit JW. Survival and death causes in differentiated thyroid carcinoma. J Clin Endocrinol Metab. 2006; 91: 313–319. 16263822 10.1210/jc.2005-1322

[6] Huang CH, Chao TC, Hseuh C, Lin KJ, Ho TY, Lin SF, et al Therapeutic outcome and prognosis in young patients with papillary and follicular thyroid cancer. Pediatr Surg Int. 2012; 28: 489–494. 10.1007/s00383-012-3054-1 22274547

[7] Huang BY, Lin JD, Chao TC, Lin KJ, Hseuh C, Tsang NM. Therapeutic outcomes of papillary thyroid cancer patients in different risk groups. Oncology. 2011; 80: 123–129. 10.1159/000328912 21677457

[8] Toniato A, Bernardi C, Piotto A, Rubello D, Pelizzo MR. Features of papillary thyroid carcinoma in patients older than 75 years. Updates Surg. 2011; 63: 115–118. 10.1007/s13304-011-0060-0 21416286

[9] Mendelsohn AH, Elashoff DA, Abemayor E, St John MA. Surgery for papillary thyroid carcinoma: is lobectomy enough? Arch Otolaryngol Head Neck Surg. 2010; 136: 1055–1061. 10.1001/archoto.2010.181 21079156

[10] Hay ID, Gonzalez-Losada T, Reinalda MS, Honetschlager JA, Richards ML, Thompson GB. Long-term outcome in 215 children and adolescents with papillary thyroid cancer treated during 1940 through 2008. World J Surg. 2010; 34: 1192–1202. 10.1007/s00268-009-0364-0 20087589

[11] Vorburger SA, Ubersax L, Schmid SW, Balli M, Candinas D, Seiler CA. Long-term follow-up after complete resection of well-differentiated cancer confined to the thyroid gland. Ann Surg Oncol. 2009; 16: 2862–2874. 10.1245/s10434-009-0592-4 19655202

[12] Scheiden R, Keipes M, Bock C, Dippel W, Kieffer N, Capesius C. Thyroid cancer in Luxembourg: a national population-based data report (1983–1999). BMC Cancer. 2006; 6: 102 16635261 10.1186/1471-2407-6-102 PMC1475873

[13] Links TP, van Tol KM, Jager PL, Plukker JT, Piers DA, Boezen HM, et al Life expectancy in differentiated thyroid cancer: a novel approach to survival analysis. Endocr Relat Cancer. 2005; 12: 273–280. 15947102 10.1677/erc.1.00892

[14] Cushing SL, Palme CE, Audet N, Eski S, Walfish PG, Freeman JL. Prognostic factors in well-differentiated thyroid carcinoma. Laryngoscope. 2004; 114: 2110–2115. 15564829 10.1097/01.mlg.0000149442.22393.e2

[15] Eichhorn W, Tabler H, Lippold R, Lochmann M, Schreckenberger M, Bartenstein P. Prognostic factors determining long-term survival in well-differentiated thyroid cancer: an analysis of four hundred eighty-four patients undergoing therapy and aftercare at the same institution. Thyroid. 2003; 13: 949–958. 14611704 10.1089/105072503322511355

[16] Storm HH, Plesko I. Survival of children with thyroid cancer in Europe 1978–1989. Eur J Cancer. 2001; 37: 775–779. 11311653 10.1016/s0959-8049(01)00010-7

[17] Sebastian SO, Gonzalez JM, Paricio PP, Perez JS, Flores DP, Madrona AP, et al Papillary thyroid carcinoma: prognostic index for survival including the histological variety. Arch Surg. 2000; 135: 272–277. 10722027 10.1001/archsurg.135.3.272

[18] Mazzaferri EL, Jhiang SM. Long-term impact of initial surgical and medical therapy on papillary and follicular thyroid cancer. Am J Med. 1994; 97: 418–428. 7977430 10.1016/0002-9343(94)90321-2

[19] Salvesen H, Njølstad PR, Akslen LA, Albrektsen G, Viste A, Søreide O, et al Thyroid carcinoma: results from surgical treatment in 211 consecutive patients. Eur J Surg. 1991; 157: 521–526. 1683575

[20] Akslen LA, Haldorsen T, Thoresen SO, Glattre E. Survival and causes of death in thyroid cancer: a population-based study of 2479 cases from Norway. Cancer Res. 1991; 51: 1234–1241. 1997164

[21] Riego-Iraeta A, Pérez-Méndez LF, Mantinan B, García-Mayor RV. Time trends for thyroid cancer in northwestern Spain: True rise in the incidence of micro and larger forms of papillary thyroid carcinoma. Thyroid. 2009; 19: 333–340. 10.1089/thy.2008.0210 19355823

[22] Instituto Nacional de Estadística. Available: http://www.ine.es. Accessed March 2013.

[23] Ministerio de Sanidad y Política Social. Estrategia en Cáncer del Sistema Nacional de Salud. Ministerio de Sanidad y Política Social; 2009 10.1080/00207590500411179.Motivational

[24] Herrero Zapatero E. Patología de los tumores de tiroides. Tumores de paratiroides In: Suárez C, Gil-Carcedo LM, Marco J, Medina JE, Ortega P, Trinidad J, editors. Tratado de Otorrinolaringología y Cirugía de Cabeza y Cuello. Editorial médica Panamericana SA; 2009 pp. 3751–3773. 10.1016/j.adengl.2013.04.025

[25] AJCC. AJCC Cancer Staging Manual. 2nd ed AJCC; 2010.

[26] Hanley JA, McNeil BJ. The meaning and use of the area under a receiver operating characteristic (ROC) curve. Radiology. 1982; 143: 29–36. 7063747 10.1148/radiology.143.1.7063747

[27] Sullivan LM, Massaro JM, D'Agostino RB Sr. Presentation of multivariate data for clinical use: The Framingham Study risk score functions. Stat Med. 2004; 23: 1631–1660. Review. 15122742 10.1002/sim.1742

[28] Ramírez-Prado D, Palazón-Bru A, Folgado-de-la Rosa DM, Carbonell-Torregrosa MA, Martínez-Díaz AM, Gil-Guillén VF. Predictive models for all-cause and cardiovascular mortality in type 2 diabetic inpatients. A cohort study. Int J Clin Pract. 2015; 69: 474–484. 10.1111/ijcp.12563 25234387

[29] Wilson PW, D'Agostino RB, Levy D, Belanger AM, Silbershatz H, Kannel WB. Prediction of coronary heart disease using risk factor categories. Circulation. 1998; 97: 1837–1847. 9603539 10.1161/01.cir.97.18.1837

[30] Schnabel RB, Sullivan LM, Levy D, Pencina MJ, Massaro JM, D'Agostino RB Sr, et al Development of a risk score for atrial fibrillation (Framingham Heart Study): a community-based cohort study. Lancet. 2009; 373: 739–745. 10.1016/S0140-6736(09)60443-8 19249635 PMC2764235

[31] D'Agostino RB Sr, Vasan RS, Pencina MJ, Wolf PA, Cobain M, Massaro JM, et al General cardiovascular risk profile for use in primary care: the Framingham Heart Study. Circulation. 2008; 117: 743–753. 10.1161/CIRCULATIONAHA.107.699579 18212285

[32] Palazón-Bru A, Martínez-Orozco MJ, Perseguer-Torregrosa Z, Sepehri A, Folgado-de la Rosa DM, Orozco-Beltran D, et al Construction and validation of a model to predict nonadherence to guidelines for prescribing antiplatelet therapy to hypertensive patients. Curr Med Res Opin. 2015;31:883–889. 10.1185/03007995.2015.1030377 25777159

[33] Hay ID, Bergstralh EJ, Goellner JR, Ebersold JR, Grant CS. Predicting outcome in papillary thyroid carcinoma: development of a reliable prognostic scoring system in a cohort of 1779 patients surgically treated at one institution during 1940 through 1989. Surgery. 1993;114:1050–1057; discussion 1057–1058. 8256208

