# Supplementary material for: Scoring System for Mortality in Patients Diagnosed with and Treated Surgically for Differentiated Thyroid Carcinoma with a 20-Year Follow-Up
Source: PLoS One. 2015 Jun 26;10(6):e0128620. doi: 10.1371/journal.pone.0128620 (PMC4482660; doi:10.1371/journal.pone.0128620)
Supplement: S1 Text — (DOC) [file pone.0128620.s001.doc]

The mobile app is available in the following stores:

- Play Store (Android).
- App Store (iPhone).

The name of this app is *Thyroid cancer mortality predictor* and it is free for all users of these operating systems.
